# Driving with Motor Neuron Disease: Disease-Specific Considerations, Multi-Domain Assessments and Support Strategies

**DOI:** 10.3390/brainsci16040408

**Published:** 2026-04-10

**Authors:** Jana Kleinerova, Jane Tully, Jasmin Lope, Ee Ling Tan, Alison Toomey, We Fong Siah, Peter Bede

**Affiliations:** 1 Computational Neuroimaging Group (CNG), School of Medicine, Trinity College Dublin, D02 PN40 Dublin, Ireland; 2 Department of Neurology, St James’s Hospital, D08 NHY1 Dublin, Ireland; 3 The Irish FTD/FTLD Network, Dublin, Ireland

**Keywords:** Amyotrophic Lateral Sclerosis, motor neuron disease, driving, primary lateral sclerosis, Spinal Muscular Atrophy, Kennedy’s disease, poliomyelitis

## Abstract

Motor neuron diseases (MNDs) encompass a clinically heterogeneous group of neurodegenerative conditions with varying impact on dexterity, mobility, decision making, respiratory and bulbar dysfunction. While consensus best-practice recommendations exist for genetic screening, diagnostic work-up, pharmacological and respiratory management, disease-specific facets of driving safety, assessment approaches and intervention strategies to support patients for safe driving have not been comprehensively reviewed. MNDs have unique, phenotype-specific clinical features, which are distinct form other neuromuscular conditions which necessitate a careful and systematic approach to evaluate driving safety. While MNDs are primarily associated with progressive motor impairment, extrapyramidal, cerebellar, cognitive, behavioural, and respiratory manifestations of the disease also affect driving safety and necessitate comprehensive driving assessments and individualised strategies to enable patients to continue to drive. The majority of existing papers focus on amyotrophic lateral sclerosis, and low-incidence MND phenotypes, such as PLS, SBMA, PPS, are glaringly understudied from a driving safety perspective despite the relatively slower progression of these conditions. Beyond the review of specific aspects of driving in MNDs, the main objective of this review paper is to raise awareness of non-motor aspects of MNDs with regard to driving safety and to explore viable strategies to support patients to maintain their independence. Despite the considerable differences in driving regulations around the globe, there are core, disease-specific aspects of MND which are universal. The careful consideration of these clinical factors, comprehensive domain-by-domain assessments, and the implementation of practical, individualised adaptations may enable patients to continue driving safely, maintain their independence and enhance their quality of life.

## 1. Introduction

**Figure 1:**
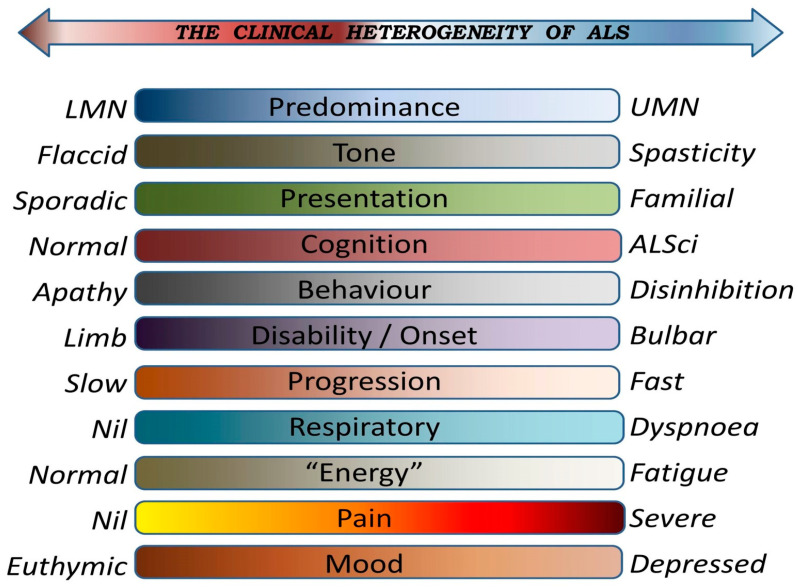
Axes of clinical heterogeneity in ALS.

Motor neuron disease (MND) is an umbrella term encompassing diverse neurodegenerative conditions with distinctive clinical features. Amyotrophic lateral sclerosis (ALS) is the most common form of MND affecting middle-aged people and typically presenting with insidious onset motor symptoms and exhibiting a rapidly progressive clinical course. ALS however is a clinically heterogeneous syndrome with considerable variations in motor disability profiles, cognitive involvement, and progression rates. The notable symptomatic heterogeneity in ALS (Figure 1) necessitates careful and comprehensive assessments in a range of clinical domains to establish patient-specific profiles early in the course of the disease to inform individualised management strategies. There are two key attributes that distinguishes ALS from the most common neurodegenerative conditions, such as Parkinson’s disease (PD), or Alzheimer’s disease (AD). ALS typically affects younger people, who are often still working, raising families, running a business, frequently travelling domestically and internationally and may be particularly reliant on driving. The other unique aspect of ALS compared to other neurodegenerative conditions, such as AD or PD, is the notable diversity of initial presentations, the striking differences in disability profiles and variability in progression rates. Some patients walk into the clinic with marked bulbar symptoms and no limb manifestations at all, while others struggle to use their limbs, but their speech and swallowing are unaffected. Some present with marked spasticity and hyperreflexia, while motor disability in others is dominated by muscle wasting and flaccid weakness. The main dimensions of disease heterogeneity in ALS includes differences in the genetic profile, site of onset, upper versus lower motor neuron predominance, cognitive and behavioural involvement, and extrapyramidal manifestations (Figure 1). Clinical and genetic overlap with other neurodegenerative conditions such as FTD/FTLD or PLS adds to the complexity of disease heterogeneity. In light of the notable differences in clinical manifestations, ALS is often conceptualised as a “spectrum disorder”. The clinical diversity of ALS is often approached along the UMN-LMN, ALS-FTD, slow–fast progressors, bulbar–spinal disability, and “familial” (genetic)–sporadic axes. While this is simplistic, it is useful to subcategorise patients into specific phenotypes, hence the generation of various patient categorisation and staging schemes based on disability profiles. The Milano–Torino staging system (MITOS), King’s staging, the Fine’til 9 (FT9) staging method, Strong criteria, and terms such as spinal–bulbar onset, are just some of the commonly used strategies to subcategorise patients with ALS [1,2,3,4,5,6,7]. The striking clinical heterogeneity of ALS (Figure 1) precludes the adoption of a single, unified management strategy and necessitates comprehensive assessments evaluating motor, extrapyramidal, cerebellar, cognitive, behavioural and social domains. Similarly to the adoption of precision pharmacological interventions and genotype-specific therapies [8,9], supportive strategies also necessitate an individualised approach based on multi-domain assessments to best support patients in their own environment, based on their current disability and in their personal social, spiritual and psychological context.

## 2. Driving with MND

Driving is surprisingly understudied in MNDs despite its unquestionable relevance to independence, employment and social interactions. While driving may seem less relevant in urban environments and locations well serviced by public transport, clinical experience and interaction with patients would suggest that it is hugely important for a significant proportion of patients with considerable quality of life ramifications. Independence, autonomy and dignity are key components of quality of life in ALS and in MNDs in general, and driving is often considered as a particularly important factor in maintaining social interactions, attending family events, engaging with the local community, attending religious or spiritual services, maintaining employment, looking after personal affairs such as banking, shopping, hair-dressing, finances, post, leisure activities, attending sporting events, going out to restaurants, bars, pubs, and, as the patients often put it, remaining an “active member of the local community”. As air travel may become increasing problematic and international travel increasingly daunting, car trips are often favoured by patients with respiratory difficulties, due to the ease of stopping when needed. Taking holidays by car and organising weekends away often provide a welcome respite from hospital attendances to relieve stress. Patients with respiratory symptoms and patients with longer symptom duration often opt for car travel and taking ferries to travel abroad instead of flying, to avoid queuing at the airports, lengthy security checks, facing flight delays and long flights in a confined space. Empirical evidence would also suggest that driving is important for psychological well-being and feeling less of a burden to family members. Beyond the social and quality of life (QoL) aspects of driving, driving is often indispensable to attend hospital consultations, clinical trial sessions, and physiotherapy, speech pathology, rehabilitation and dietetics consultations. While seldom evaluated formally, the inability to drive and residing in the countryside may impact on the frequency of hospital attendances and willingness to participate in pharmacological trials and research studies and may lead to social isolation. It is also noteworthy that travelling in a car as a passenger may also be challenging even if driven by someone else, as access, seating, getting out of the car, and communication may become problematic or uncomfortable. Expert review, adjustments for individual disability profiles are therefore also paramount for non-driving car passengers. The emotional impact of driving cessation is relatively well researched [10,11] and it has been extensively studied in older individuals. It is a notoriously difficult transition as driving is often consider as an integral factor of one’s independence [12] and may have self-esteem ramifications. Family support, caregiver network and support in the community play a crucial role to make this transition less troublesome. In a condition where a substantive literature has been generated for presymptomatic screening [13], diagnostic work-up, disease staging [2], spasticity management, genetic counselling, pharmacological management, multidisciplinary interventions [14], respiratory support, feeding tube placement, palliative interventions, machine learning applications, etc. [15], it is curious how driving in MND is glaringly under-researched despite its considerable “real-life”, practical ramifications and quality of life implications. The objective of this paper is therefore the careful review of motor and non-motor aspects of MNDs and their potential impact on driving, the suggestion of a comprehensive domain-by-domain assessment scheme and the discussion of practical strategies to enhance patient independence.

## 3. Methods

A narrative review has been conducted to review existing studies, assessment recommendations and driving adaptations in motor neuron diseases. The search terms “MND”, “ALS”, “PLS”, “Polio”/”Poliomyelitis”, and “Kennedy’s disease”/”SBMA” were individually paired with “Driving” on PubMed. Information on study design (prospective, retrospective, multicentre, etc.), patient cohort (ALS, SBMA, PPS, etc.), number of participants, main objectives, clinical instruments and assessment batteries, interventions, and main study findings were retrieved from the identified studies. To provide a context for multi-domain assessments, motor and extra-motor manifestations of MNDs are also briefly reviewed, focusing on recently published data. Only original articles published in English were considered, and opinion pieces, editorials, reviews, and meta-analyses were excluded. In light of the paucity of relevant papers identified, a narrative review format has been adopted instead of a “systematic review” to highlight study shortcomings, knowledge gaps, and research priorities.

## 4. Results

### 4.1. Motor Manifestations of ALS

ALS presents with varying degrees of comorbid lower (LMN) and upper motor neuron (UMN) dysfunction (Figure 1). The most common clinical manifestations of LMN involvement is flaccid paresis with muscle bulk loss, fasciculations, often leading to poor grip, selective hand muscle involvement with the preferential involvement of the first dorsal interosseous (FDI) [16], wrist-drop, finder-drop, head-drop, foot drop, proximal muscle weakness and respiratory weakness [17]. Wrist-drops, foot-drops and head-drops are typically managed by the careful fitting of orthoses, chin lifts, splints, etc. High-resolution biomechanical measures in gait studies reveal considerable ankle dysfunction with reduced range of motion and plantarflexion strength [18]. This not only affects walking stability, but interferes with driving. It is important to highlight that despite their disability, patients with early stage ALS perform similarly to controls on simulated driving tasks [19]. Therapeutic exercise, particularly low-to-moderate-intensity aerobic and resistance training, may help preserve muscle strength and function in ALS and slow functional decline, potentially supporting safer driving for longer [20,21]. UMN dysfunction manifests as spasticity, cramping, and clonus, but the resulting clinical picture depends on the proportion of UMN/LMN dysfunction in a specific body region. Spasticity in ALS stems from upper motor neuron dysfunction and leads to progressive muscle stiffness, reduced mobility, difficulty with activities of daily living (ADLs), and driving [22]. Spasticity often contributes to fatigue and pain [23,24]. First-line treatments for spasticity in ALS include oral antispasmodic drugs, such as baclofen or tizanidine, with botulinum toxin type A (BTX-A) injections and physiotherapy. BTX-A combined with physiotherapy has shown short-term improvements in muscle function without significant side effects [22]. Moderate-intensity endurance exercise may also reduce spasticity, and improve motor and even pulmonary function [25]. Dexterity is often affected early in the course of the disease in limb-onset forms of ALS. A notable asymmetry is often observed in the earlier stages with one limb much more affected than the other, providing an opportunity for adaptive strategies to perform daily tasks including hand controls for driving or, rarely, left-foot accelerators. Bulbar motor manifestations of ALS include dysarthria, dysphagia and pseudobulbar affect, progressively impacting communication, swallowing and social functions [26,27]. Bulbar dysfunction also contributes to excessive drooling (sialorrhea) which may be distracting while driving. Sialorrhea often necessitates careful pharmacological interventions which in turn may impact on alertness and concentration. Lower limb dexterity and bulbar dysfunction all impact driving, as they may interfere with getting in and out of the car, steering, operating switches, gears, and pedals, keeping the head upright and moving the head left and right. Discomfort from cramping, pain from adhesive capsulitis, weight loss, and drooling are all common symptoms in ALS which may impact on concentration. Spasticity may reduce fine control of pedals and gears. Dysarthria may impact on making calls and engaging with voice commands and satellite navigation. Sudden emotional responses, especially in those experiencing pseudobulbar affect or pathological crying and laughter (PCL) may also be distracting [28]. While classically conceptualised as a “pure” UMN-LMN condition, solely driven by primary motor cortex [29] and spinal anterior horn degeneration [30], it is increasingly clear that extrapyramidal, cerebellar, and sensory components contribute to impaired fine motor control in ALS [31,32,33,34,35,36]. Cerebellar, sensory and extrapyramidal facets of ALS are notoriously overlooked [31,32,33,34,37]. The degree of cerebellar pathology is difficult to evaluate clinically due to coexisting UMN/LMN dysfunction dominating the clinical picture, but a series of recent neuroimaging papers have confirmed cerebellar cortex, deep cerebellar nuclear and cerebro-cerebellar connectivity alterations with a predilection to specific cerebellar regions in both ALS [36,37] and PLS [38,39]. Cerebellar pathology in MNDs has widespread clinical implications beyond fine motor control; due to its diverse physiological roles, it may adversely impact on judging distance, perception of speed, gaging size and dimensions, and emotional responses. It is known to contribute to pseudobulbar affect, and has numerous cognitive and behavioural correlates [40,41,42,43,44,45,46,47,48,49,50,51]. Subtle extrapyramidal manifestations have also long been noted in ALS [52]. Post mortem and neuroimaging studies have consistently highlighted basal ganglia degeneration in both ALS and PLS [53,54,55,56], and there is also evidence of both structural and functional corticobasal connectivity alterations [57]. It is therefore increasingly clear that extrapyramidal motor deficits contribute to motor impairment in MNDs [57,58,59]. While the core clinical features of ALS are widely known, there is relatively limited awareness of subtle coexisting proprioceptive, extrapyramidal and cerebellar deficits and these factors all impact on motor control, gait, dexterity and bulbar function. Accordingly, motor deficits in ALS should not be solely assessed from a UMN/LMN perspective and sensory components, proprioceptive deficits, motor integration circuits, basal ganglia degeneration and cerebellar dysfunction should also be considered [33,34]. While involuntary movements are not classically associated with ALS, they are occasionally reported and may impact on driving. These may include rest minipolymyoclonus, thumb tremors, pseudodystonic thumb posture, action minipolymyoclonus, and action tremors. Minipolymyoclonus, or polyminimyoclonus, is a low-amplitude, high-frequency, arrhythmic, jerky involuntary movement, often seen in the hands and fingers in ALS but also observed in other lower motor neuron disorders such as Spinal and Bulbar Muscular Atrophy (SBMA) or Spinal Muscular Atrophy (SMA) [60,61]. Action tremor is also observed at times and corresponds to electromyography peak frequency [62].

### 4.2. Non-Motor Features of ALS/MND

**Figure 2:**
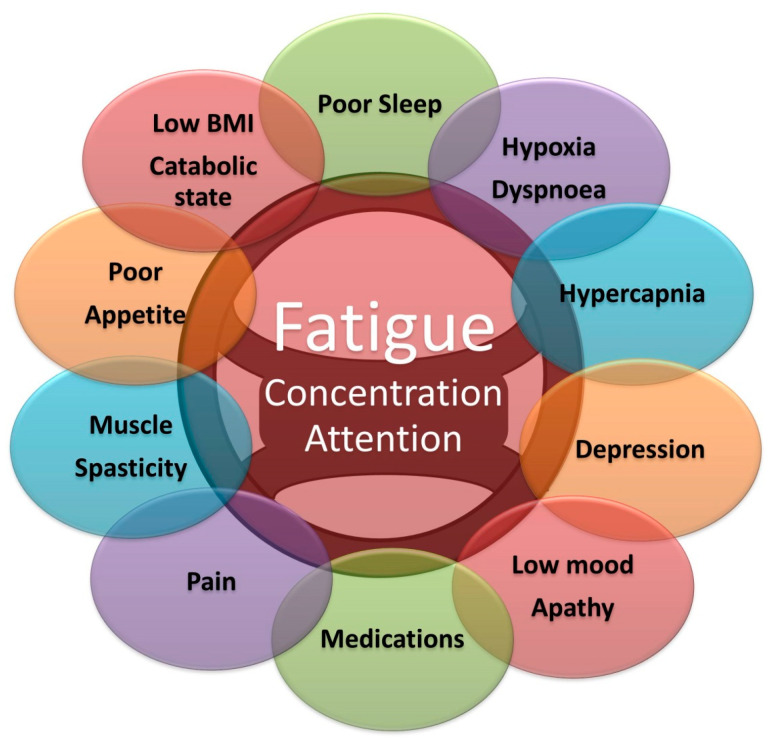
Components of fatigue in MNDs and opportunities for intervention.

Adding to the complexity of motor manifestations, a range of extra-motor manifestations may impact on activities of daily driving in ALS and other MNDs. Cognitive deficits [63], behavioural disturbances [64], fatigue [23], apathy [65], and respiratory weakness [66] are just some of the clinical facets of ALS which may impact on driving safety. Neuropsychological deficits in ALS are traditionally linked to executive dysfunction [67] and behavioural deficits [68,69], but recent studies have confirmed a wider spectrum of neuropsychological manifestations including memory [70,71,72] and language deficits [73,74,75], deficits in social cognition [76,77], and apathy [65]. Impulsivity, disinhibition [78], impaired decision making and risk ascertainment, deciphering the intentions of others [77,79,80], and poor concentration all have obvious ramifications for driving safety [81]. Data from other neurodegenerative conditions confirm that disinhibition and executive dysfunction are associated with increased driving errors [82]. The neuropathological substrate of these deficits has been extensively investigated in ALS [83,84,85], but the modifying effects of cognitive reserve, education, cognitive rehabilitation and medications on neuropsychological manifestations are also increasingly recognised [14,86,87,88]. There is a range of other ALS-associated non-motor manifestations which may impact on driving such as discomfort from weight loss, poor tolerability of longer drives, sialorrhea, somnolence, dyspnoea, morning headaches, and fatigue [23]. Understanding of the causes of both motor and non-motor manifestations is crucial so that the appropriate adaptive strategies can be implemented. Somnolence and poor concentration are also commonly reported in ALS and have an obvious impact on driving. Fatigue in ALS and other MNDs is multifactorial [23,89], arising from a combination of hypoxia, hypercapnia, sedating medications, low mood, fragmented sleep, etc. (Figure 2). Commonly used medications in ALS, such as baclofen, anticholinergics prescribed for drooling, opiate analgesia, and benzodiazepines prescribed for spasticity, are all notoriously sedating in isolation and especially in combination. A systematic approach for evaluating somnolence and fatigue allows targeted interventions such as the introduction of non-invasive ventilation (NIV), medication adjustments, etc., which may improve these symptoms. Evidence for effectively treating fatigue in ALS is very limited. Pharmacological and non-pharmacological interventions show possible, but largely unproven, benefits [23,90]. Possible pharmacological therapies include Modafinil [91] and 3-4 diaminopyridine (DAP), with only mild improvement in subjective fatigue scores [92]. Fatigue is recognised as the leading cause of fatal road traffic accidents (RTAs) worldwide and has been linked to functional connectivity alterations [93]. Sensory dysfunction is not typically associated with MNDs even though paraesthesiae are often reported by patients. Recent clinical [94,95,96,97], neuropathology [98,99,100,101], neurophysiology [102,103,104,105], spinal and brain imaging studies [103,106,107,108] have all highlighted varying degrees of primary sensory and sensory processing deficits [109]. These are thought to include subclinical proprioceptive deficits and suggest evidence of spinal posterior column degeneration [103,110]. Weight loss in ALS has been traditionally exclusively linked to bulbar dysfunction, but recent studies have identified complex neuroendocrine- and hypothalamus-mediated network alterations [111,112]. Significant weight loss and catabolic state may influence driving comfort, mood, concentration, and also contribute to a sense of generalised fatigue (Figure 2).

Respiratory weakness is a hallmark of ALS, leading to reduced ventilatory function, weak cough, ineffective secretion clearance, and eventual respiratory failure. This decline is primarily driven by diaphragmatic and intercostal muscle denervation and results in reduced maximum inspiratory/expiratory pressures [113,114]. Impaired respiratory function impacts on concentration, decision making, and cognitive function [115,116]. Data from non-ALS cohorts indicate that even mild increases in CO_2_ can impair decision making and problem-solving, and may cause fatigue, headaches, and dizziness [117,118]. Respiratory muscle training and expert physiotherapy may improve quality of life, but effects on overall lung function are variable [114,119]. The negative impact of hypercapnia on cognitive and psychomotor function is well established [120] and this is increasingly prevalent in ALS as the disease progresses [121]. Individuals with hypercapnia perform worse on tasks measuring vigilance and processing speed and logical memory tests compared to non-hypercapnic OSA patients [122]. The mainstay of therapy is non-invasive ventilation (NIV). Improvement in hypercapnia-associated symptoms can be typically noted soon after NIV initiation. Early introduction of NIV is associated with better outcomes [123,124,125].

Sleep disorders are also highly prevalent in ALS and are multifactorial. Respiratory muscle weakness, pain, psychological factors, existential distress, and impaired sleep regulation are all contributory factors. The most common presentations include insomnia, nocturnal hypoventilation (NH), obstructive sleep apnoea (OSA), restless legs syndrome (RLS), excessive daytime sleepiness (EDS), and, less commonly, rapid eye movement sleep behaviour disorder (REMSBD). In patients with restrictive lung disorders, sleep-related hypoventilation associated with hypercapnia may affect approximately 30% of patients and is typically linked to diaphragmatic, intercostal, and accessory respiratory muscles weakness [126,127,128,129]. Sleep disturbances worsen physical and mental health during the day, and are associated with faster progression rates and reduced survival, especially when coupled with respiratory dysfunction [130]. Untreated OSA confers a 1.5 to 4 times higher risk for RTAs, and RTA risk is thought to be commensurate with OSA severity. While OSA is typically treated by Continuous Positive Airway Pressure (CPAP) [131,132], BIPAP/NIV with pressure support is the preferred intervention in ALS [66]. Insomnia is typically driven by a combination of physical symptoms and psychological distress, and it may raise RTA risk twofold [126,128,133,134]. Sleep deprivation reduces vigilance, even without subjective sleepiness, and impairs multitasking [132]. Excessive daytime sleepiness (EDS) at the wheel is associated with an increased risk of accidents, with an odds ratio (OR) of 2.51 in some studies [135]. EDS may manifest in diminished attention, poor concentration, slower reaction times, and inappropriate line crossing. In recognition of this association, the automobile industry has recently developed a range of safety systems such as lane departure warning, eye-tracking, steering wheel grip detection, back-up cameras, 360° camera systems, park-assist technologies, traffic sign and traffic light recognition sensors, radar systems to maintain a safe distance, and various collision avoidance systems, such as automated braking and steering corrections [136]. The Intelligent Drowsiness and Fatigue Recognition (IDFR) system, for example, employs a customised convolutional neural network (CNN) algorithm for ocular tracking [137]. These systems often integrate data from a multitude of sensors and feed real-time data into complex machine learning (ML) models. Input physiological and behavioural markers typically include eye and mouth movements, brain activity markers, heart rate variability, head posture, steering behaviour, and lane deviation. Neuromorphic vision systems based on on-board camera data achieve excellent accuracy in detecting driver drowsiness [138,139].

From a driving perspective, it is very important that note that not every neuronal circuit and functional network is affected in ALS and that several functions such as vision, hearing and many parietal processes are relatively preserved. ALS is not associated with global atrophy, but it selectively affects specific brain regions, with a predilection to the primary motor cortex, corpus callosum, and descending corticospinal tracts. Depending on the phenotype, certain frontotemporal regions are also affected. Occipital and parietal brain regions however are relatively spared [140] and, while some visual processing networks may be eventually affected [141], frank visual field defects are never experienced by patients. Similarly, while some auditory regions are eventually involved [106], hearing loss and hearing impairment are not typically experienced in ALS.

### 4.3. Non-ALS MND Phenotypes

**Figure 3:**
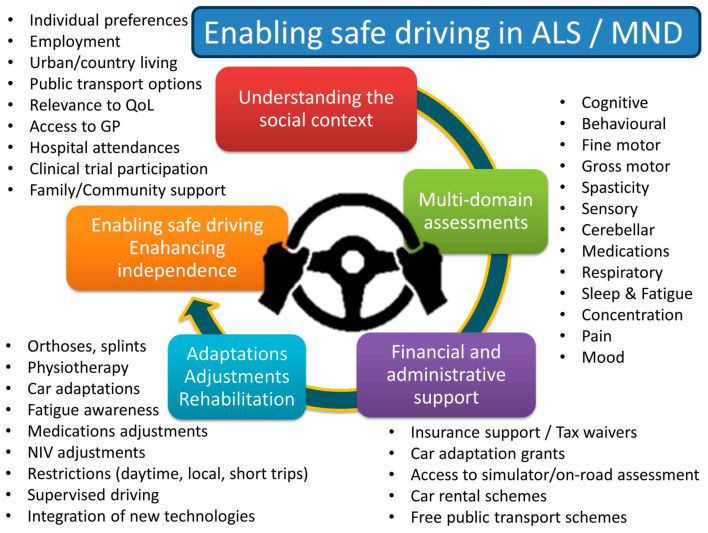
Assessment and intervention strategies for safe driving in MNDs.

The vast majority of best-practice recommendations in motor neuron diseases solely focus on ALS as the most common form of MND. MNDs, however, encompass a clinically diverse spectrum of conditions, each posing unique management challenges with regard to driving. Primary Lateral Sclerosis, or PLS, is a progressive neurodegenerative condition which manifests with progressive limb spasticity and pseudobulbar manifestations and is primarily dominated by upper motor neuron degeneration. Limb stiffness, decreased rapid finger movements, difficulty getting in and out of the car, and spasticity slowing pedal operation are just some of the motor sequelae of UMN degeneration in PLS. In addition to progressive primary motor cortex and corticospinal tract degeneration in PLS [142], recent studies have demonstrated cognitive deficits [143,144] and subcortical [55,145], cerebellar [39], and frontotemporal involvement [143,146,147], contributing to the extra-motor and extrapyramidal manifestations of the disease [85,148]. These observations highlight that PLS is not a “pure” UMN disorder and clinical assessments need to explore extra-motor domains. Spinal and Bulbar Muscular Atrophy (SBMA), a.k.a. Kennedy’s disease, is another progressive multi-system neurodegenerative condition which primarily manifest as a lower motor neuron (LMN) disorder. It is associated however with a range of other neurological, cardiac, metabolic and endocrine features. Glucose intolerance, type II diabetes, hyperlipidaemia, sensory neuropathy, postural tremor, obstructive sleep apnoea, painful muscle cramps and myalgia, breathlessness, cognitive manifestations, repolarisation abnormalities and ensuing arrhythmias are just some of the disease-specific clinical factors that need to be carefully weighted up when assessing driving safety [149,150]. Post-poliomyelitis syndrome (PPS) typically manifest decades after the initial infection, and while assessments typically focus on limb control and strength, a range of extra-motor manifestations are often reported in PPS, including fatigue, somnolence, poor concentration, pain and discomfort [89]. A systematic approach (Figure 3), exploring all disease-specific clinical facets of these conditions, is therefore paramount to assess driving safety in these MND subtypes. Progressive muscular atrophy (PMA) is a low-incidence, LMN-predominant motor neuron disease phenotype, which is associated with longer survival than ALS, and typically presents with relentless muscle wasting and fasciculations. Recent studies have demonstrated some degree of extra-motor manifestations including mild cognitive impairment [151,152].

### 4.4. Driving Studies in MND

There is a striking paucity of studies addressing driving safety in MND; these are summarised in Table 1. The most commonly administered instruments in these studies are the Montreal cognitive assessment (MoCA); the ALS Cognitive Behavioural Scale (ALS-CBS); gait speed (m/s); ALS Functional Rating Scale—revised total score (ALSFRS-r); and simulated driving assessment with a Lane Change Task [19,153]. Studies using driving simulation tasks show that individuals with mild-to-moderate ALS generally perform similarly to healthy controls under cognitive or visual distractions. However, in motor distraction tasks, the ALS group performed significantly slower [154]. Safe driving requires complex motor skills, especially when multitasking or when distracted. The impact of cognitive impairment on driving safety is glaringly understudied and poorly characterised in ALS. A recent longitudinal study identified no direct association between baseline cognitive function and driving status 4 months later [155], suggesting that current cognitive performance is not a predictor of future driving ability. ALSFRS-r scores however have been suggested to predict driving cessation [156]. Longitudinal tracking of ALSFRS-r scores over at least 24 weeks reveals a drop in both fine and gross motor scores and exhibits a non-linear trajectory of functional decline [157]. Motor impairment and driving capacity is more apparent under distraction and may unmask motor vulnerabilities not evident during undistracted driving. Driving assessment methods in these studies included driving simulation, computer simulation and also the modified questionnaire of the Norwegian ParkWest study [158]. The Lane Change Task used in simulation studies quantifies perception, lane change quality, and lane-keeping ability, with secondary tasks designed to detect at-risk drivers. Evidence regarding how driving ability declines across the stages of disease remains glaringly limited [19,154,155]. The driving literature of non-ALS MNDs is even more limited. No prospective purpose-designed studies have specifically evaluated the driving performance of patients with PLS, despite the considerable literature on motor disability in PLS [147,159,160] and the emerging literature on the neuropsychological [85,143,144], cerebellar [38] and extrapyramidal [56] facets of the disease. Similarly, no study has been specifically dedicated to the disability profiles of patients with SBMA, and only generic recommendations have been suggested for this cohort, such as on-road assessments with standardised routes to determine driver competence [161]. There have been no studies dedicated to PMA, which classically presents with progressive LMN dysfunction and gradual respiratory involvement [162]. PPS is known to impair driving ability in up to 70% of those affected [163], but Swedish data indicate that with adequate support, up to 57% of patients can continue to drive safely. Common car adaptations in this cohort include hand controls, hand braking and accelerating systems, and steering wheel knobs [164,165]. Some studies highlight the positive impact of continued driving on self-esteem [166]. Reliance on assistive devices for mobility and activities of daily living (ADLs) is negatively associated with driving [167]. A 10-year cohort study of patients with lower limb disability, including poliomyelitis patients, revealed that only 0.6% of patients had road traffic accidents (RTAs) due to disability and confirmed that drivers with physical disabilities are not at an increased risk of RTAs [168]. Individualised interventions, such as modern ankle–foot orthoses (AFOs) in polio patients, are associated with improved automobile driving [169]. Data from questionnaires on driver disability, adaptations and involvement in RTAs showed no difference in accident rates from drivers in the general population [170].

### 4.5. Lessons from Other Neurological Conditions

Parkinson’s disease (PD) is another neurodegenerative condition which is associated with a range of both motor and cognitive symptoms, but it has a much higher incidence than ALS and the specific impact of motor and non-motor manifestations on driving safety is much better characterised [172,173,174]. It has been associated with indecisiveness at T-junctions and reduced rear-view and side mirror usage. Standard clinical measures of PD were not predictive of actual driving performance [175]. Contrary to the limited impact of physical disability on driving in polio, early stages of dementia are associated with a higher risk of failing performance-based road tests and impaired driving abilities [176]. Neuropsychological testing in older individuals revealed that driving performance may be linked to three key cognitive domains: speed of processing, visuospatial abilities, and memory. A comprehensive neuropsychological assessment is necessary to accurately determine the risks of unsafe driving [177,178]. There is a consensus in the literature that comprehensive neuropsychological testing is required in any neurodegenerative syndrome as part of an integrated driving assessment [178,179,180]. The Trail Making Test (TMT), Symbol Digit Modalities Test, and Purdue Pegboard Test have shown predictive value for driving performance in neurological populations. Combined neuropsychological, driving simulator and clinical assessments have shown the most accurate prediction for fitness to drive, with an overall accuracy of 92.7% [181,182,183,184]. Driving assessment strategies in the dementia literature include Lane Change Task (LCT) [154], on-road assessments [185], driving simulator evaluation [186], Standard New Zealand licencing testing and the advanced driver assessment [187]. Driving in multiple sclerosis (MS) has been linked to more accidents, slower reaction times, and lower Driving Safety Scores (DSSs). Impaired driving performance in MS has been linked to cognitive deficits such as impaired spatial short-term memory, working memory and selective attention, translating into a higher number of accidents [182]. In summary, lessons from other neurological conditions suggest that cognitive performance, rather than motor performance, predicts driving safety. Disease-specific, motor-function focused rating scales are poor predictors of driving safety.

### 4.6. Assessment Strategies in ALS/MND

A systematic (Figure 3) domain-by-domain approach is necessary to examine driving safety in ALS and other MNDs, as seen in Table 2. It is crucial to fist explore the social context and the importance attached to driving in individual patients. Assessments for driving safety should first consider phenotype-specific motor disability profiles, such as UMN versus LMN dysfunction predominance, disease stage, fine and rapid movements, spasticity, and the ability to control steering, pedals, and main driving controls. In light of the emerging evidence of multi-system cerebral involvement in ALS, PLS, and SBMA (see above), clinical assessment should also be expanded to uncover additional cerebellar, extrapyramidal and proprioceptive deficits. Given the considerable extra-motor features of most MNDs (see above), executive function, behavioural profiles, mood, medications, fatigue, somnolence, medications, respiratory function, and cardiac function (SBMA) should also be systematically evaluated. A number of disease-specific cognitive and behavioural instruments have been developed and validated in ALS in recent years, which can be administered and interpreted with ease by non-neuropsychologists either in person or remotely [68,188,189,190,191,192,193,194]. Other cognitive assessment schemes have been used to predict on-road driving ability in other neurological conditions: the Visual Object and Space Perception battery (VOSP) [195], the Behavioural Assessment of the Dysexecutive Syndrome (BADS) [196] and the Rookwood Driving Battery (RDB) [197]. A poor score on the VOSP is linked to worse performance in simulated driving scenarios. The BADS screens for executive impairments in problem solving, cognitive flexibility and temporal judgement. An RDB with a score over 10 has 88% positive predictive value for failing an on-road assessment. Medications, doses, interaction, synergistic effects, and time of administration need to be carefully reviewed by either an experienced neurologist or pharmacologist. There is compelling evidence that commonly prescribed drugs in ALS adversely affect driving performance, such as anti-spasticity medications, anticholinergics, and TCAs, but benzodiazepines [198], opiates [199,200], SSRIs [201,202], and cannabis [203,204], and their various combinations [202,205], pose a particular risk for driving safety and response times. Respiratory function needs to be evaluated as per relevant guidelines [66] and NIV settings adjusted if indicated. Fatigue, somnolence and sleep disorders need to be explored using the appropriate clinical questions or questionnaires used in OSA.

### 4.7. Interventions

**Figure 4:**
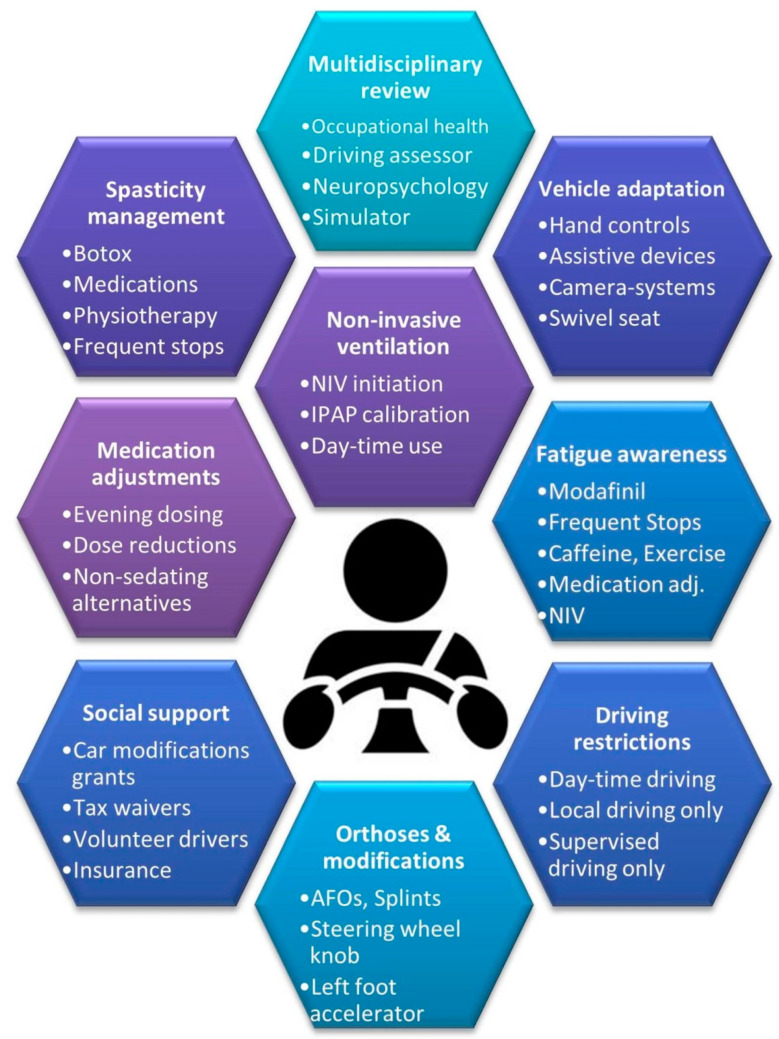
Safety optimisation strategies for driving with ALS/MND.

Interventions should always be individualised following a comprehensive multi-domain assessment to address the identified deficits in specific domains (Figure 4). The optimisation of respiratory support may include nocturnal and/or additional daytime non-invasive ventilation, the careful adjustment of IPAP (Inspiratory Positive Airway Pressure) and EPAP (Expiratory Positive Airway Pressure) settings, airway and secretion clearance with breath-stacking and the use of cough-assist machines. Medications, salivary gland botulinum toxin injections or salivary gland radiotherapy (RT) maybe used to dry up distracting secretions and sialorrhea. Medication adjustments may improve somnolence, improve concentration and alleviate fatigue. Frequently used anti-spasticity medications, such as baclofen, tizanidine or benzodiazepines, are notorious to contribute to fatigue and drowsiness; therefore, dose reduction or evening administration should be considered. Commonly prescribed tricyclic antidepressants (TCA), such as Amitriptyline, and tetracyclic antidepressants (TECA), such as Mirtazapine, are also sedating and should be administered in the evening when no further driving is planned. Opiate analgesia, administered *per os* (PO), as a patch or via a syringe driver, and anticholinergic medications for sialorrhea (Scopolamine patches, Glycopyrrolate, etc.) are all sedating and may cause drowsiness. It is noteworthy that most patients with ALS are on a combination of the above medications, often with synergistic side-effect profiles contributing to drowsiness. The attentive review of medications is therefore paramount when assessing suitability for driving in MND. Practical considerations, such as only driving during the day, in familiar environments, accompanied by a friend or family member, or only driving for short distances, should be considered. Patient-tailored, disability-specific adaptations (Figure 4), such as push–pull hand controls, steering knobs, and swivel seats, may enable patients with MND to operate their vehicles independently. Personalised adaptations should always reflect a patient’s individual disability profile. In patients with lower limb disability, push/pull lever devices are often considered to allow both acceleration and braking control with just one hand. Electronic, fly-by-wire “trigger accelerators” requiring simple finger flexion and extension, and “ghost ring accelerators” requiring side-to-side finger movements, are just some of the solutions implemented. In patients with limited right leg mobility, a left-footed accelerator can be installed to the left of the brake pedal. In patients with impaired hand function, a steering wheel knob is often attached to the steering wheel and so that the car to be steered with just one hand. Additionally, 360-degree swivelling seats are often installed for easier access. Voice-activated or touch-based secondary controls are sometimes fitted for lights and wipers. While not a common practice, in some jurisdictions, vehicles can be modified to allow drivers to remain in their wheelchairs while driving [206,207,208,209]. Recent innovations in driverless car technology promise to increase levels of independence for people with disabilities [185]. Many governments offer various tax reduction schemes, and financial and grant support for adaptive car modifications. Frequent breaks and naps are commonly recommended with patients with respiratory compromise and they have been shown to significantly reduce RTAs [210]. The European Respiratory Society (ERS) formed an OSA task force and recommended an Apnoea–Hypopnea Index (AHI) threshold of >15 events/h for driving restriction [211].

### 4.8. Governing Concepts

The priority of any of the above assessments (Figure 3) and interventions (Figure 4) is to enhance the independence, dignity and autonomy of people with motor neuron diseases. Initial discussion around driving has to commence by exploring the patient’s own views on their driving safety and the importance of driving to them. Driving safety assessments need to be comprehensive, with the evaluation of motor (hand and lower limb function), bulbar (sialorrhea and communication), cognitive (decision making, visuospatial skills, distractibility, and concentration), and behavioural (apathy and disinhibition) function, sedative medication and drug combinations (anti-spasticity, analgesia, and anticholinergics), respiratory function, sleep, and fatigue, as shown in Table 2. By focusing on the patient’s own preferences (importance of travel and social interactions), social circumstances (residence, town/country, financial means, and family support), and clinical profiles (bulbar/spinal and UMN/LMN predominance), patient-centred, individualised interventions need to be designed and implemented by a multidisciplinary team.

### 4.9. Stakeholders

Most national health authorities issue expert driving guidelines for people with neurological conditions including temporary bans following neurosurgery, stroke, traumatic head injury, seizures, etc. Particularly detailed disease-specific guidelines typically exist for epilepsy, dementia, and patients with movement disorders, but most countries do not have MND-specific driving regulations or best-practice regulations. In most European countries the neurologist, physiotherapist and occupational therapist have an initial discussion with the patient about their driving preferences and their own views regarding driving safety. This is typically followed by an expert on-the-road assessment. Many insurance companies mandate the notification of new diagnoses and an opinion from the patient’s general practitioner (GP) or specialist with regard to driving safety. Based on the multidisciplinary assessment, a letter is often issued to the GP with regard to driving and a consensus opinion is then communicated to the insurance company. An on-the-road driving assessment is often requested from a licenced driving instructor or evaluator, which can be extremely helpful as constructive suggestions are provided based on observations under real-life driving conditions. National charities and patient advocacy groups are often actively involved in the assessment and support of patients in the community. UK Driver and Vehicle Licensing Agency (DVLA) guidelines provide detailed procedural guidance for either a new or a worsening notifiable medical condition [185]. The DVLA needs to be notified by the individual, and a medical and driving assessment subsequently arranged, and special restriction may then be introduced. In Ireland, the National Driver Licence Service (NDLS) and insurance company must be formally informed. Communication between regulatory bodies, insurance companies, funding agencies and health care professionals (GPs, OTs, and neurologists) is often complex, strained, fragmented and lengthy. In a rapidly progressive condition, a simple, transparent and streamlined process is desirable. Only very limited data exist on insurance costs for patients with specific conditions [212] and many would argue that such costs should be subsidised.

### 4.10. Knowledge Gaps and Future Directions

In addition to the strikingly low number of driving safety studies in MND, research is limited to ALS and PPS cohorts. The sample sizes of the identified studies were generally small and samples were particularly small for patients with mild-to-moderate motor disability [19], as shown in Table 3. The impacts of respiratory involvement and sleep disorders are under evaluated. Clinical data in many of the identified studies are scarce and the sedating effect of prescribed medication combinations is often overlooked. Assessments typically primarily focus on motor function. Current assessment batteries for driving fitness in motor neuron diseases are not standardised and disease-specific instruments are seldom utilised. A variety of tools have been implemented in recent studies, including the Edinburgh Cognitive and Behavioural ALS Screen (ECAS) [68], the Montreal cognitive assessment (MOCA) [213], the ALS Cognitive Behavioural Scale (ALS-CBS) [188] and the revised ALS Functional Rating Scale [214] (ALSFRS-r). There is a notable lack of on-road driving evaluation under specific driving conditions (rain, snow, night time, and motorway) and longitudinal follow-up on performance. The limited literature and experience from other neurological conditions would suggest that driving should be systematically evaluated, simulator-based screening should be considered and on-the-road testing should be carried out under specific driving conditions. Assessments should be performed by medical practitioners, neuropsychologists, occupational therapists, and driving instructors [215]. Comprehensive domain-by-domain assessments should be performed (Table 2) and any incidents or near-misses, including minor parking accidents, getting lost, anxiety attacks, and abandoning the car and asking for assistance, should be recorded and registered so that future driving safety decisions can be informed based on real-life data and feedback. Prospective studies need to be designed to specifically explore the determinants of safe driving across cognitive, behavioural, respiratory, sleep and motor domains. The impact of continued driving and driving cessation on the social, professional, recreational, mental health, and medical aspects of an individual affected by MDN should also be systematically studied. The QoL implications for driving cessation should also be evaluated in ALS/MND.

In the absence of an international consensus regarding driving recommendations for ALS/MND, dedicated satellite meetings should be held at large international meetings to address this important practical gap in MND care. A multitude of well-attended international meetings dedicated to ALS/MND take place annually, often also attended by patients and their caregivers, which may be ideal platforms for such discussions. The annual ALS/MND symposium organised by the UK MNND association, the annual ENCALS meeting organised by the European network to cure ALS, FILSLAN, the French health network for rare diseases, focusing on ALS and motor neuron diseases, and NEALS—Network of Excellence for ALS are just some of the large organisation holding annual meetings attended by a large group of experts from around the globe. Expert committees should be formed with the representation of relevant stakeholders, patients, physiotherapies, occupational therapies, patient advocacy groups and charities to discuss the principles of multi-domain driving safety assessments and consensus strategies to enhance driving with MND. Draft guidelines could then be circulated internationally and refined based on feedback. Such international documents could then serve as the basis of national guidelines. There are also valuable learning opportunities from more common neurological conditions such as multiple sclerosis, acquired spinal injuries, stroke and dementia syndromes [216]. Large prospective studies specifically exploring driving preferences, barriers and effective interventions should be conducted and published to shape future guidelines. Research groups and clinical centres should capitalise on remote assessment tools and connected devices to measure fatigue, respiratory function, and bulbar and limb function remotely in real time. With the relevant data regulations in place, this data could be interpreted by neurologists and physiotherapists remotely and they could advise patients and families regarding driving safety. No patient should have to face increased premiums for driving with a disability. Patient advocacy groups and neurologists engaging in MND care should encourage discussions with insurance companies and government agencies to advocate for tax reliefs, insurance fee reductions and car adaptation grants to support patients with MND and their families. Machine learning (ML) models have been successfully used in nearly all aspects of ALS/MND research to accurately classify individual patients into relevant diagnostic, phenotypic and prognostic categories or disease clusters [217,218,219,220,221,222,223,224]. Based on the success of recent ML initiatives, it is conceivable that clinical data from individual patients may be used to predict driving safety if large, well-designed data repositories are generated to assess, establish and validate the determinants of driving safety in ALS/MND. Driving simulators have been successfully used for assessments in some countries, and while this does not replace on-the-road assessment, it may be an ideal initial screening tool for driving safety. The implementation of new technologies, such as voice command-based navigation, semi-autonomous driving, camera systems, fatigue monitors, and advanced collision avoidance systems may facilitate driving with a significant disability.

## 5. Conclusions

In light of the complex multi-domain disability profile of ALS and other MNDs, there is an urgent and unmet need to study driving safety with the involvement of relevant stakeholders and generate evidence-based, consensus, best-practice recommendations for driving assessments and adaptations. Communication between government agencies, health care professionals and insurers needs to be streamlined. The governing principle behind such initiatives is to enhance driving safety, maintain independence, support patient autonomy, and improve the quality of life of people living with ALS and various MNDs.

## Figures and Tables

**Table 1:** Overview of research studies on driving in MND.

Authors & Year	Cohort	Study Design	Number of Participants	Main Focus & Objectives	Clinical Data and Instruments	Main Study Findings and Conclusions
Hayes et al., 2020 [19]	ALS	Prospective	28 ALS 20 Controls	Driving capacity	MOCA, ALS-CBS, gait speed, ALSFRS-r, LCT	LCT scores between pALS and HC are not different under motor, cognitive, or visual distraction. Driving assessment needs to be expanded longitudinally.
Hayes et al., 2016 [153]	ALS	Prospective	30 ALS 20 Controls	Driving simulation tasks & driving skills	LCT, MOCA, ALS-CBS, gait speed, ALSFRS-r	pALS with mild cognitive and motor deficits perform similarly to HC. Individuals typically cease driving within 2 years but objective indicators are lacking.
Hayes et al., 2016 [154]	ALS	Prospective	20 ALS 9 Controls	Driving capacity while distracted using computer simulation	Gait speed, MOCA, TMTB, LCT, MDT, VDT	pALS perform poorly under motor distraction.
Taule et al., 2025 [155]	ALS	Observational study	31 ALS	Impact of cognitive change on driving cessation	ECAS, ALSFRS-r	Cognitive function is not a predictor of driving cessation.
Hayes et al., 2019 [156]	ALS	Prospective	27 ALS 20 Controls	Clinical correlates of driving capacity	ALSFRS-R, LCT, MDT, VDT	Distraction variables and ALSFRS-r predict driving cessation.
Lings, 1991 [171]	HSP	Prospective	52 Paraparesis 109 Controls	The impact of paresis & spasticity on driving	Grip strength, RT	Paresis affects reaction times more than spasticity.
Khan et al., 2024 [163]	PPS	Cross-sectional	200 PPS	Challenges faced in PPS	Post-Polio Clinic Questionnaire	Pain, fatigue, and muscular weakness reported by 91.5%; driving deemed impossible by 70%.
Selander et al., 2020 [164]	Polio	Retrospective	145 Polio	Outdoor mobility with polio	Mobility, independence, pain, depression, mobility, transport questionnaire	In total, 57% independent and active drivers. Dependence for outdoor mobility linked to depression.
Zeilig et al., 2012 [167]	Polio	Retrospective	123 Polio	Social and functional barriers in poliomyelitis	Demographics, B-ADL, E-ADL, mobility	LSP impacts on employment as per ICF.
Ysander, 1966 [168]	Polio	Cohort study	494 Polio	RTAs in patients with poliomyelitis	Disability profiles	Successful vehicle modifications for LEoP, low % (0.6) of RTAs due to disability.
Steinfeld et al., 2003 [169]	Polio	Retrospective	55 Polio	Benefits of modern AFOs in polio	AFO acceptance, functional capacity, comfort	Benefits of carbon fibre orthoses: improved ADLS, ambulation and driving.
Henriksson et al., 2004 [170]	Polio	Cross-sectional	793	Safety of drivers with disabilities	Driving questionnaire, adaptations, safety, involvement in RTAs	Benefits of vehicle adaptation, 1 out of 10 drivers involved in RTAs over 3.5 years.

**Table 2:** A domain-based systematic assessment strategy for MND/ALS.

Assessment Domain	Specific Factors to Consider
Social context	Individual driving preferences, employment, habitation (town/country), relevance to QoL, frequency of hospital attendances, clinical trial participation, community support, isolation, etc.
Cognition	Executive function, visuospatial skills, spatial memory, attention, concentration
Behaviour	Disinhibition, apathy, social cognition
Mood	Anxiety, depression, outlook, motivation
Medications	Anti-spasticity meds, anticholinergics, opiates, benzodiazepine, SSRI, SNRI, TCA, antihistamines, cannabis, syringe drivers, patches
Pain	Spasticity, adhesive capsulitis, cramps, pressure sores, odynophagia, oral candidiasis
Extra-motor manifestations	Proprioceptive, extrapyramidal, cerebellar manifestations, paraesthesia, sialorrhea, pseudobulbar affect
Involuntary movements	Polyminimyoclonus, thumb tremor, involuntary crying and laughter
Tone	Spasticity, cramps
Fatigue	Somnolence, concentration, attention
Sleep	OSA, Hypoxic events, REM sleep behaviour disorder, restless legs syndrome
Respiratory function	Morning headaches, orthopnoea, hypercapnia, NIV-dependence
Fine motor control	Dexterity, ankle–foot control
Gross motor control	Pedal and steering operation, ability to get in and out of the vehicle, wheelchair use
Sensory examination	Proprioceptive deficits, sensory ataxia, pseudoathetosis, paraesthesia, vibrotactile deficits
Financial and regulatory contexts	Insurance premium, availability of car modification grants, charity support, government support, free travel on public transport, car tax waiver

**Table 3:** Knowledge gaps and research priorities for safe driving in MND.

Knowledge and Research Gaps	Priorities and Future Directions
Absence of disease-specific guidelines & best-practice recommendations	Prospective studies & accident rate registries
Small, poorly designed, retrospective studies	Predictors and prognostic indicators for driving cessation need to be studied
Limited clinical instruments implemented	Outcome assessment of driving restrictions (local, daytime, morning only, etc.)
Focus on motor function primarily	International expert committees for driving with MND
Overlooking cognitive and behavioural aspects of the disease	Satellite meetings at large international conferences
Generic disability-based regulations and guidelines	Raising awareness of MND-associated challenges with decision makers, insurance companies, local driving authorities
Limited access to simulators and on-road assessments	Campaigning at local health authorities, governments, insurance industry for subsidies and grants
Long waiting times for assessments	Prompt access to simulators and road tests
Unclear coordination of care	Access to timely car adaptations
Slow approval of car modification grants in many jurisdictions	Renting schemes of modified vehicles
Clinicians have a low threshold of advising driving cessation	Financial grants for renting and adaptations
Social context, QoL implications often overlooked	Volunteer driver network to access hospital appointments
The impact of commonly administered medications in ALS seldom considered	Involvement of relevant stakeholders: patients, caregivers, families, charities, patient advocacy groups
Poor access to neuropsychology	Implementation of new technologies, drive-by-wire, touch screens, voice command, collision avoidance systems, back-up & 360° camera systems, park-assist technology, semi-autonomous driving, etc.
Blanket driving cessation recommendations instead of restriction such as daytime, local, morning driving	Consideration of experience from other neurological conditions MS, PF, AD, MCI, etc. Establishment of MND-specific assessment and car adaptation schemes
Limited ongoing research despite huge practical relevance	Collection of patient perspectives and caregiver perspectives regarding driving experience

## Data Availability

No new data were created or analysed in this study.

## Glossary

ABG: arterial blood gas, AD: Alzheimer’s disease, ADLs: activities of daily living, AFO: ankle–foot orthoses, AHI: Apnoea–Hypopnea Index, ALS: amyotrophic lateral sclerosis, ALSbi: ALS with behavioural impairment, ALS-CBS: ALS Cognitive Behavioural Scale, ALSci: ALS with cognitive impairment, ALSFRS-r: revised ALS Functional Rating Scale, ALS-FTD: ALS with frontotemporal dementia, ALSnci: ALS with no cognitive impairment, B-ADL: basic activities of daily living, BADS: Behavioural Assessment of the Dysexecutive Syndrome, BTX-A: botulinum toxin type A, CNN: convolutional neural network, CPAP: continuous positive airway pressure, DAP: 3-4 diaminopyridine, DSS: Driving Safety Score, DVLA: Driver and Vehicle Licencing Agency, E-ADL: extended activities of daily living, ECAS: Edinburgh Cognitive and Behavioural ALS Screen, EDS: excessive daytime sleepiness, ENCALS: the European network to cure ALS, EPAP: expiratory positive airway pressure, ERS: European Respiratory Society, FDI: first dorsal interosseous, FILSLAN: Filière de Santé Maladies Rares SLA, FT9: Fine’til 9, FTD: frontotemporal dementia, FTLD: frontotemporal lobar degeneration, GP: general practitioner, HC: Healthy controls, HD: Huntington’s disease, ICF: International Classification of Functioning, Disability, and Health, IDFR: Intelligent Drowsiness and Fatigue Recognition System, IPAP: inspiratory positive airway pressure, LCT: lane change task, LEoP: late effects of poliomyelitis, LMN: lower motor neuron, LSP: longstanding poliomyelitis, MDT: multidisciplinary, MDT: motor distraction task, MITOS: the Milano–Torino staging system, ML: machine learning, MND: motor neuron disease, MOCA: Montreal cognitive assessment, MS: multiple sclerosis, NDLS: National Driver Licence Service, NEALS: Network of Excellence for ALS, NH: nocturnal hypoventilation, NIV: non-invasive ventilation, NP: neuropsychology, OR: odds ratio, OSA: obstructive sleep apnoea, OT: occupational therapy, pALS: patients with ALS, PBA: pseudobulbar affect, PCL: pathological crying and laughter, PCV: pressure-controlled ventilation, PD: Parkinson’s disease, PLS: primary lateral sclerosis, PO: per os—by mouth, Polio: patients with prior poliomyelitis, PPS: post-poliomyelitis syndrome, PSV: pressure support, PT: physiotherapy, QoL: quality of life, RDB: Rookwood Driving Battery, REMSBD: rapid eye movement sleep behaviour disorder, RLS: restless legs syndrome, RT: Reaction time, RT: radiotherapy, RTA: road traffic accident, SALT: speech and language therapy, SBMA: spinal and bulbar muscular atrophy, SMA: spinal muscular atrophy, SP: speech pathology, TCA: prescribed tricyclic, TECA: tetracyclic antidepressants, TMT: Trail Making Test, TMTB: Trail Making Test Part B, TOSCA: transcutaneous tcpCO_2_ and SpO_2_ monitoring system, UMN: upper motor neuron, VDT: visual distraction task, VOSP: Visual Object and Space Perception battery, WHO: World Health Organisation.
